# Unusual sites of bone involvement in Langerhans cell histiocytosis: a systematic review of the literature

**DOI:** 10.1186/s13023-020-01625-z

**Published:** 2021-01-02

**Authors:** Nahid Reisi, Pouran Raeissi, Touraj Harati Khalilabad, Alireza Moafi

**Affiliations:** 1 Department of Pediatric Hematology and Oncology, Faculty of Medicine, Child Growth and Development Research Center and Isfahan Immunodeficiency Research Center, Seyed Al-Shohada Hospital, Isfahan University of Medical Sciences, Isfahan, Iran; 2 Department of Health Services Management, School of Health Management and Medical Information Science, Iran University of Medical Sciences, Tehran, Iran; 3 Department of Health Economics, School of Health Management and Medical Information Science, Iran University of Medical Sciences, Tehran, Iran

**Keywords:** Langerhans cell histiocytosis, LCH disease, Bone lesion, Rare disease, Children, Adults, Systematic review

## Abstract

**Background:**

Langerhans cell histiocytosis (LCH) is a rare disease that originates from the uncontrolled proliferation and accumulation of bone marrow-derived immature myeloid dendritic cells. Dendritic cells are a type of histiocyte that play an important role in the human immune system and are found in the bone, skin, stomach, eyes, intestines, and lungs.

**Objective:**

This systematic review aimed to collect and report published case reports of rare bone disease caused by LCH to avoid misdiagnoses or delays in diagnosis.

**Methods:**

We systematically searched Scopus, PubMed, Embase, and Web of Sciences from August 1, 2000 to December 31, 2019. Studies reporting cases of LCH with rare bone involvement were included.

**Results:**

We identified 60 articles including 64 cases. Of the identified cases, 31 (48.4%) involved children, and 33 (51.6%) involved adults. Additionally, 46.9% (30 individuals) were from Asian countries. The mean age of the children was 7.6 ± 4.3 years and that of the adults was 36 ± 12 years. The findings indicated that unifocal bone involvements were the most prevalent form of the disease (68.7%), and, overall, the skull and chest wall were the most commonly affected bones in both adults and children. The spine and long bones were the second most commonly affected bones in children, and the spine and jaw were the second most commonly affected bones in adults. Pain and swelling were the most frequent presenting signs among the investigated cases, and loss of consciousness, myelopathy, nerve palsy, visual loss, torticollis and clicking sounds were rare signs. Osteolytic lesions were the most frequent radiologic feature (62.5%), and intracranial hemorrhage, fluid–fluid level, dura and intracranial extension and pathologic fractures were rare radiological features. Total excision, curettage and observation in the unifocal group of patients and systemic chemotherapy in the other groups (i.e., multifocal and multisystem) were the most frequent management approaches. The recovery rates of the unifocal and multifocal groups were 77.3% and 81.8%, respectively, while that of the multisystem group was 55.5%. The rates of recurrence and mortality in the multisystem group were 11% and were higher than those in the other groups.

**Conclusions:**

LCH is a rare disease that can affect any organ in the human body. However, bone is the most commonly involved organ, and rare bone involvements may be the first or only symptom of the disease due to the rarity of such lesions; a lack of familiarity with them may result in misdiagnosis or delayed diagnosis.

## Introduction

Langerhans cell histiocytosis (LCH) is a rare disease with a prevalence of 2–5 cases per million in children and 1–2 cases per million in adults annually [1, 2]. The etiology and pathogenesis of the disease remain unclarified; earlier theories stated that LCH is a reactive process, but recent findings based on the identification of oncogenic *BRAF* or *MAP2K1* mutations in most cases of LCH suggest that it is a clonal neoplasm and originates from the uncontrolled proliferation and accumulation of bone marrow-derived immature myeloid dendritic cells [3–6]. LCH predominantly affects children ages 5 to 15 years, with a mean age of 3 years [2, 3] and is more prevalent in male cases than in female cases [2, 6]. Its presentation in neonates and adults is rare [7] and in those older than 50 years is an exception [8].

Abnormal dendritic cells can infiltrate any organ, and, depending on the number and site of involved organs, the disease was previously classified as unifocal LCH with a solitary bone lesion (eosinophilic granuloma), multisystem with bone involvement, diabetes insipidus, exophthalmos (Hand–Schüller–Christian disease) and disseminated involvement with risk organ dysfunction (Abt–Letterer–Siwe syndrome) [9]. Recently, based on the revised classification of histiocytoses, LCh has been subclassified as follows: single-system LCH, lung LCH and multisystem LCH with or without risk organ involvement (liver, spleen, and bone marrow) [10]. The most common form of the disease is single-system LCH of the bone (approximately 75–80%) [3, 10], which can manifest as a uni- or multifocal form [2, 6]. The unifocal form is more common than the multifocal form.

LCH can affect any bone, but more than 50% of bone lesions belong to the skull, ribs and pelvis. The auditory ossicles, inner ear, sphenoid wings, clivus, and the short tubular bones of the hands and feet are rarely involved [9]. Pain, swelling, soft tissue mass, and, occasionally, bone deformity and bone fracture are symptoms of isolated bone involvement, but systemic symptoms are less common [11].

The diagnosis of bone lesions is based on imaging studies and histopathological examination. Radiography is the gold standard of imaging studies, and computed tomography (CT), magnetic resonance imaging (MRI), bone scintigraphy, positron emission tomography–computed tomography (PET–CT) and whole-body MRI are complementary imaging studies. The most common radiologic feature of the bone involvements is a lytic lesion that may present as a moth-eaten, punched-out, geographic or expansile form [9]. Destructive bone lesions or erosive forms are less common. Histopathological examination and immunohistochemical staining (with CD1a, S100 protein, and/or CD207 antibodies) of tissue samples are necessary to confirm bone lesions [5, 9].

The treatment and outcome of bone lesions depend on the extent and severity of the disease. Unifocal bone involvements are the least severe form of the disease and are usually treated locally by excision, curettage, intralesional steroid injection or radiation therapy [6, 9]. The outcome of such treatment has been good, and more than 80% of the patients recover completely [5, 6, 11]. However, multifocal bone involvements and multisystem disease with bone involvements require systemic or combination therapies and their prognosis depends on the site of bone involvement and accompanying involved organs. Multisystem involvement with “risk organs” has the worst prognosis [5, 9].

Because of the relative rarity of LCH, we conducted this systematic review to collect and identify clinical presentations, methods of diagnosis, treatment and outcomes of rare cases of LCH with bone involvement to avoid misdiagnoses or delays in diagnosis.

## Method

### Search strategies and information sources

In this study, the rare osseous involvement of LCH in children and adults was reviewed. A literature search was conducted in Scopus, PubMed, Embase, and Web of Sciences from August 01, 2000 to December 31, 2019. Appropriate English keywords such as “Langerhans cell histiocytosis”, “Hand-Schueller-Christian syndrome”, “bone”, “skull”, “case report”, and other similar keywords were used to search the articles in the databases. To identify any remaining studies, we hand-searched the bibliographies of all the included studies, relevant review articles, and the internet. The full search strategy is available in the “Appendix”.

### Eligibility criteria

All the case reports on rare cases of LCH disease that described and reported bone involvement and were published in the English language were included in our search regardless of patient age. Case reports that did not document (I) demographic characteristics, (II) site(s) of involvement, (III) symptoms and signs, and (IV) method of diagnosis were omitted from the list of the articles. Reviews and meta-analyses, proceeding articles, editorials and letters, news articles, posters, conference articles, book chapters, poorly described cases or case series without primary data or without a description of individual patient data and articles without author names were also excluded.

### Study selection

In the first step, the stated data sources were searched by the third author (Touraj Harati Khalilabad) and the extracted articles were imported into the reference management software ENDNOTE. After removing duplicates, the remaining articles were screened by first author (Nahid Reisi) using the title and abstract information. In the second step, the full text of the articles was checked by two trained authors (Alireza Moafi and Pouran Raeissi) to assess their inclusion. Each article was reviewed by two individuals independently, and if the authors had opposing opinions about an article, the first reviewer (Nahid Reisi) evaluated the article.

A “rare case” of LCH in this review was defined as follows:

I, B*one involvement in unusual locations* such as the auditory ossicles and internal ear, clivus, sphenoid bone with involvement of the clivus, sphenoid wings or pituitary stalk [9], clavicle, sternum, forearm, fibula, short tubular bones of hand and foot, and lesions crossing a cranial suture [9, 12–15].

II, A*typical radiographic appearance* such as epiphyseal lesions, transphyseal lesions, extracranial “button sequestrum”, posterior vertebral arch lesions of the spine, fluid–fluid levels on CT or MRI, cortical bone lesions, extension of a lesion into the dura or brain substance, and pathologic fractures [12, 13], and/or extradural hematoma of skull LCH [9].

III, U*nusual presentations* such as neurologic manifestations of spinal disease resulting from vertebral collapse and impingement or from extradural extension of the lesion [12] and cranial nerves palsy [9].

IV, Unusual age—the neonatal period [7] and age older than 50 years [8].

### Data extraction

In the final step, the required parameters and characteristics of each study were extracted and included author names, year, country of origin of the published article, demographic characteristics of the cases, symptoms and signs, sites of involvement, lesion types, method of tissue sampling and diagnosis, strategies for management and patient outcomes.

## Results

**Fig. 1:**
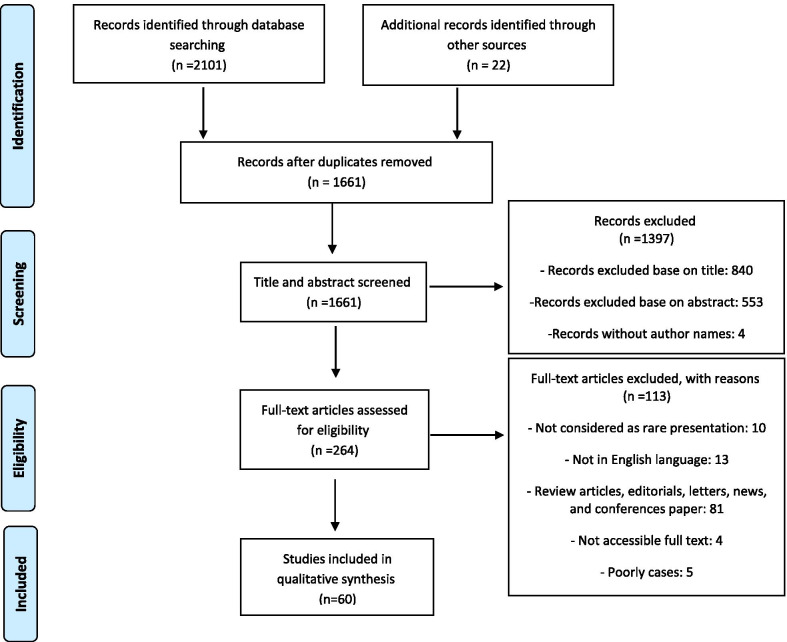
PRISMA flow diagram of literature search

The results of the initial search of databases and hand searching other sources yielded 2,101 and 22 articles, respectively; after eliminating duplicates, 1,661 articles remained. After screening the articles by title and abstract, 264 articles remained, and the remainder were excluded because they were not relevant. After reviewing the full text of the 264 articles, 113 articles that lacked eligibility information were excluded. Finally, 60 articles encompassing 64 rare cases met the eligibility criteria to enter the study [2–4, 6–8, 14–67]. Figure 1 shows the flow diagram of the literature search.

### Demographic characteristics

Sixty articles were included in the final analysis, comprising 64 cases. Most of the case reports (71.7%) were published between 2010 and 2019 and were from Asian countries. More than two-thirds (70.3%) of the cases were male, and the male-to-female ratio was 2.3. Thirty-one cases (48.4%) were children (younger than 18 years), and the remainder (51.6%) comprised adults (older than 18 years). The mean age of the children was 7.3 years (SD = 4.3 years) with an age range of 5 months to 16 years, and the mean age of the adults was 36 years (SD = 12 years) with an age range of 18–66 years

Based on the number of involved bones and other organ involvement, the cases were classified into three categories: unifocal bone involvement, multifocal bone involvement, and multisystem. Unifocal bone involvement had the highest frequency among the other groups of bone involvement and accounted for 68.7% of the cases.

In general, the skull and chest wall showed the highest rate of involvement (42.2% and 23.4%, respectively) followed by the spine and pelvis (9.4% each). Short tubular bones were involved in 3% of the cases. The demographic and clinical characteristics of the included cases are shown in Table 1.

**Table 1:** The demographic and clinical characteristics of rare cases of bone involvement (n = 64)

Publication decade	N (%)
2000s	17 (28.3)
2010s	43 (71.7)
Region	
Asia	30 (46.9)
Europe	19 (29.7)
America	12 (18.7)
Africa	2 (3.1)
Oceania	1 (1.6)
Gender	
Male	45 (70.3)
Female	19 (29.7)
Age (years)	
Less than 1	2 (3.2)
2–18	29 (45.2)
19–50	28 (43.8)
More than 50	5 (7.8)
Category	
Unifocal	44 (68.7)
Multifocal	11 (17.2)
Multisystem	9 (14.1)
Site of bone involvement	
Skull	27 (42.2)
Facial bones^a^	4 (6.2)
Spine	6 (9.4)
Chest wall^b^	15 (23.4)
Pelvis	6 (9.4)
Long bones	4 (6.2)
Short tubular bones	2 (3.1)
Mean age (years)	Mean ± SD (n)
Children (Age range in children)	7.6 ± 4.3 (n = 31)
Adults (Age range in adults)	36.4 ± 12 (n = 33)

^a^Mandible, maxilla, nasal bones and orbit

^b^Rib, clavicle, sternum and scapula

### Symptoms and signs

Table 2 depicts the symptoms and signs of the included cases. Localized pain (48.4%) and swelling (26.5%) were the most frequent presenting symptoms and signs, followed by headache (15.6%), hearing loss (12.5%), localized mass (9.4%) and limitation of motion (9.4%).

**Table 2:** Symptoms and signs of rare bone involvements of LCH cases (*n* = 64)

	n (%)
Symptom and sign	
Localized pain	31 (48.4)
Swelling ± pain	17 (26.5)
Headache ± vomiting	10 (15.6)
Hearing loss	8 (12.5)
Mass ± tenderness	6 (9.4)
Limitation of motion	6 (9.4)
Loss of consciousness	4 (6.2)
Vertigo	4 (6.2)
Visual loss	3 (4.7)
Torticollis	3 (4.7)
Tinnitus	2 (3.1)
Otalgia	2 (3.1)
Otorrehea	2 (3.1)
Numbness	2 (3.1)
Weakness	2 (3.1)
Nerve palsy	1 (1.5)
Quadruparesis	1 (1.5)
Paresthesia	1 (1.5)
Hyposthesia	1 (1.5)
Disequilibrium	1 (1.5)
Nystagmus	1 (1.5)
Proptosis	1 (1.5)
Chest tightness	1 (1.5)
Clicking sound	1 (1.5)
Teeth mobility	1 (1.5)
Gingival dehiscent	1 (1.5)
Afternoon fever	1 (1.5)

Hearing loss, otalgia, otorrhea, tinnitus, vertigo, nystagmus and disequilibrium were symptoms and signs of temporal bone involvement with extension and destruction of the inner ear. Left otitis media with effusion and drainage of cerebrospinal fluid (CSF) after ventilation tube insertion was a rare presentation of mastoid involvement with destruction of the posterior wall of the external auditory canal with extension to the sigmoid sinus. Hearing loss in both ears for 10 years treated with corticosteroids and worsening following the discontinuation of corticosteroids was another sign of mastoid involvement.

Headache, vomiting, loss of consciousness, swelling and soft tissue mass (progressive or tender with or without pain) were symptoms and signs of calvarium bone involvements with intracranial hemorrhage. Facial puffiness and eye proptosis was a sign of maxillary sinus involvement. Rapidly progressing visual loss occurred following optic canal involvement (1 case) and gradual visual loss following petrous apex involvement (2 cases). Bilateral abducent nerve palsy was also another sign of petrous apex involvement.

Symptoms of cord compression, including pain, weakness, numbness, paresis and paresthesia, were observed in 3 cases with spinal involvements and 1 case of sacral spine (S1) involvement with extension into the neuroforamina. Torticollis was also reported in cases with cervical spine or occipital condyle involvement.

The other rare symptoms in these cases were chest tightness, clicking sound (due to pathological fracture of the clavicle), teeth mobility and gingival dehiscence. Fever was also reported in one case with sternum involvement.

### Sites of bone involvement

**Fig. 2:**
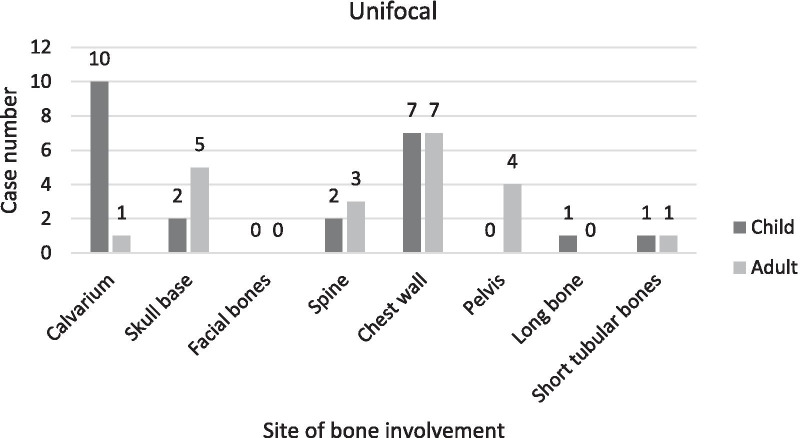
Sites of bone involvement in adults and children in unifocal sub-group

**Fig. 3:**
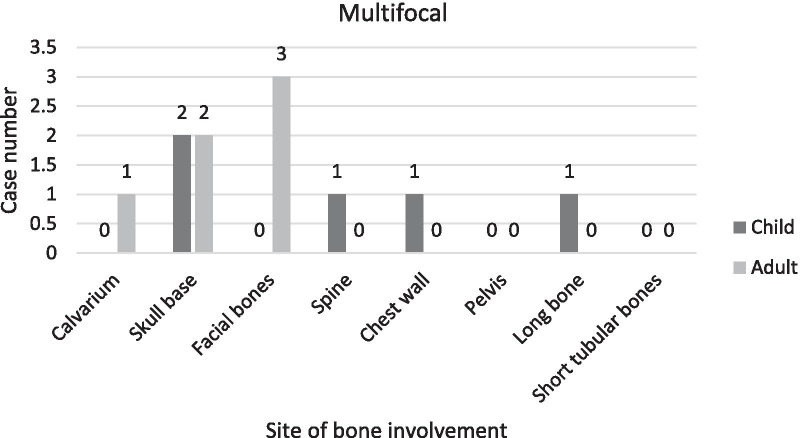
Sites of bone involvement in adults and children in multifocal sub-group

**Fig. 4:**
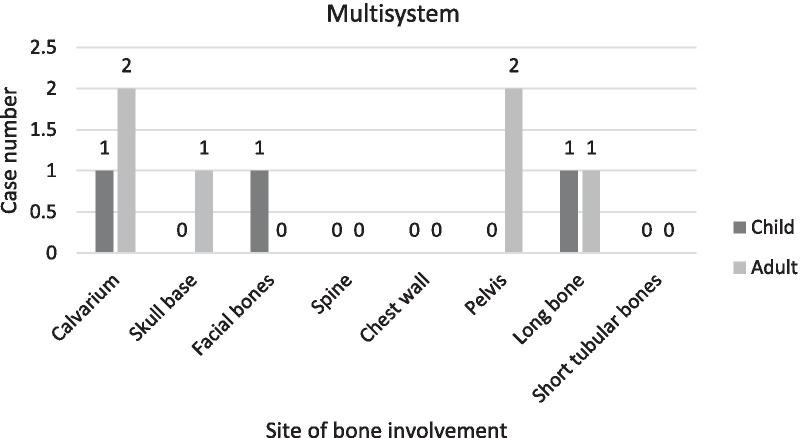
Sites of bone involvement in adults and children in multisystem sub-group

The findings indicated that most of the cases showed unifocal bone involvement (68.75%). Multifocal and multisystem bone involvements were observed in (17.19%) and (14.06%) of the cases, respectively. The sites of bone involvement in children and adults for the three stated categories are presented in Table 3 and Figs. 2, 3, 4.

**Table 3:** Sites of bone involvement in children and adult LCH cases (n = 64)

Site of bone involvement	Child			Adult		
	Unifocal	Multifocal	Multisystem	Unifocal	Multifocal	Multi system
Calvarium	10	0	1	1	1	2
Skull base	2	2	0	5	2	1
Facial bones	0	0	1	0	3	0
Spine	2	1	0	3	0	0
Chest wall	7	1	0	7	0	0
Pelvis	0	0	0	4	0	2
Long bone	1	1	1	0	0	1
Short tubular bones	1	0	0	1	0	0
	23	5	3	21	6	6

**Fig. 5:**
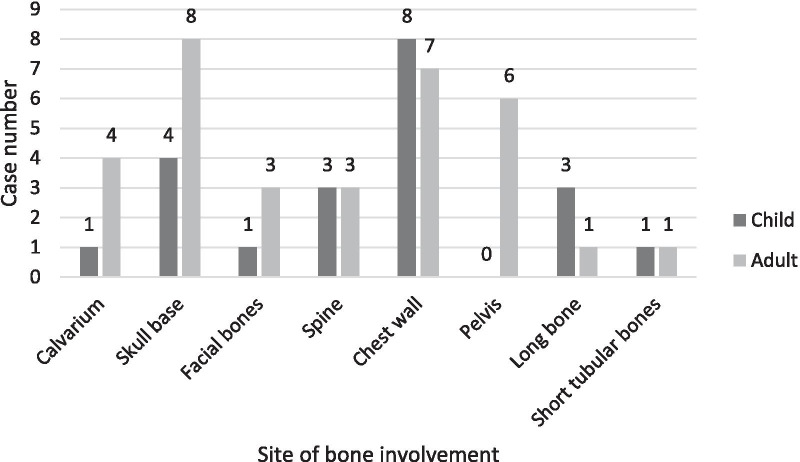
Sites of bone Involvement in adults and children

Of the 64 identified cases, 31 were children and 33 were adults; the prevalence of the disease did not differ between the two groups based on our findings. For the two groups, the most common affected bones were as follows: in children: skull (48.4%), chest wall (22.6%), spine and long bones (each 9.7%); in adults: skull (36.4%), chest wall (21.2%), spine (9.1%) and jaw (6.1%). TPelvic bone involvement was observed exclusively in adults, and involvement of the short tubular bones of the hand and foot did not differ between the two age groups (Table 3 and Fig. 5).

In the skull, calvarium involvements were more prevalent than in the skull base, and mostly involved children. However, the parietal and occipital regions were the most affected sites, and intracranial hemorrhage was exclusively observed in children. Skull base involvements, which mostly affected the inner ear, were more prevalent in adults (Table 3).

### Imaging studies, type of bone lesions and tissue sampling methods

Table 4 summarizes the imaging studies, types of bone lesions and tissue sampling methods of all the cases.

**Table 4:** Imaging studies, type of lesions and tissue sampling methods of LCH bone lesions in cases (*n* = 64) of case reports

	n (%)
Imaging study	
Plain radiograph	30 (46.9)
CT scan	47 (73.4)
MRI	46 (71.9)
Bone survey	14 (21.9)
Bone scintigraphy	22 (34.4)
PET/CT	10 (15.6)
Type of lesions	
Osteolytic^a^	40 (62.5)
Destructive	11 (17.2)
Expansile lytic	8 (12.5)
Erosive	5 (7.8)
Tissue sampling method	
Surgical biopsy	28 (43.7)
Closed biopsy	12 (18.7)
Open biopsy	11 (17.2)
Needle biopsy	6 (9.4)
CT-guided biopsy	5 (7.8)
Curettage biopsy	1 (1.6)
Sampling was not removed	1 (1.6)

^a^In 1case fluid–fluid level [29] and in another case "button sequesterum" [8] was seen

The most common types of imaging studies used in all the patient groups were CT (47 cases; 73.4%) and MRI (46 cases; 71.9%), followed by plain radiography (46.9%), bone scintigraphy (34.4%), bone survey (21.9%) and PET-CT (15.6%).

Radiologically, osteolytic lesions, with a prevalence of 62.5% (n = 40), were the most common bone involvement among the cases, followed by destructive, expansile lytic and erosive forms, in that order. In a 66-year-old man with a parietal osteolytic lesion, a sequestered fragment within the lesion (button sequestrum) was reported. Another study reported a 4 year old with LCH discrete fluid–fluid levels within the epidural component.

The findings indicated that, in 98.4% (n = 63) of cases, the diagnoses of bone LCH was confirmed by histopathological and immunohistochemical examination on tissue sampling of the lesion. In one case, tissue sampling of the lesion was not performed, and the diagnosis was merely made based on the clinical background of LCH and imaging examinations. Surgical biopsy (43.7%) and closed biopsy (18.7%) were the most frequent methods of sampling.

### Management approach and outcomes

Table 5 summarizes the treatment modalities used to treat the LCH bone lesion patients, along with the outcomes.

**Table 5:** Management and outcome of bone lesions in cases (*n* = 64) of case reports

Management	n (%) / involved sites			References
	Unifocal (n = 44)	Multifocal (n = 11)	Multisystem (n = 9)	
Total excision	18 (40.9)/skull, spine, sternum	2 (18.2)/temporal + intracranial extention + sigmoid sinus, + mandible, Both jaws	–	[8, 16, 18, 20, 22–31, 40, 42, 52, 57]^a^
Total excision + chemotherapy	2 (4.5)/spine, rib		1 (11.1)/petrous + SCC + middle cranial base	[34, 38, 64]
Subtotal excision + chemotherapy	1 (2.3)/skull	1 (9.1)/petrous + inner ear + sphenoid	1 (11.1)/bilateral mastoid + C2	[6, 19, 59]
Subtotal excision + radiotherapy	1 (2.3)/spine	1 (9.1)/clivus, sella + sphenoid sinus		[33, 60]
Radiosurgery	1 (2.3)/skull	–	–	[17]
Radiotherapy	1 (2.3)/spine	1 (9.1)/temporal + clivus + occipital + atlantoaxial joint	–	[32, 54]
Radiotherapy + epidural steroid injection	1 (2.3)/ [51]	–	–	[51]
Systemic chemotherapy	2 (4.5)/clavicle, skull	4 (36.3) /odontoid process + C2 + femur, bilateral petrous + bilateral labyrinth, both jaws + petrous apex + otic capsule + clivus, sternum + greater trochanter + tibia	3 (33.3)/bilateral parietal + both jaws, maxillary sinus, Ilium + T6 + 6th rib	[7, 21, 53, 55, 58, 61, 62, 65, 66]
Chemotherapy + radiotherapy	–	–	1 (11.1)/temporal with involving dura	[3]
Analgesics, + intralesional steroid injection + chemotherapy	–	–	1 (11.1)/both femur	[66]
Curettage	9 (20.5)/clavicle, sternum, humerus, acetabulum, metatarsal	–	–	[14, 36–38, 41, 43, 49, 50]^b^
Curettage + radiotherapy	2 (4.5)/sternum, metacarpal	–	–	[15, 45]
Observation	4 (9.0)/sternum, clavicle, scapula, ilium	–	–	[4, 39, 44]
Conservative	2 (4.5)/scapula, spine	1 (9.1)/radius, ulna	1 (11.1)/femur	[2, 35, 46, 63]
Not determined	–	1 (9.1)/both jaws	1 (11.1)/sacral	[56, 67]
Outcome after initial treatment			n (%)	
Complete recovery without complication	33 (75.0)	6 (54.5)	3 (33.3)	
Complete recovery with permanent complication	1 (2.3)	3 (27.3)	2 (22.2)	
Recurrence	1 (2.3)	1 (9.1)	1 (11.1)	
Lost follow- up	1 (2.3)	0 (0.0)	0 (0.0)	
Not determined	8 (18.1)	1 (9.1)	2 (22.2)	
Death	0 (0.0)	0 (0.0)	1 (11.1)	

SCC, semicircular canal; C2, second cervical spine; T6, sixth thoracic spine

^a^ 3 cases, ^b^ 2 cases

In the unifocal group, total excision (40.9%), curettage (20.5%) and observation (9%) were the most frequent modalities applied to treat the patients, followed by “excision plus chemotherapy”, “curettage plus radiotherapy”, “systemic chemotherapy”, and “conservative management” (4.5% each). However, “subtotal excision plus radiotherapy” or “sub-total excision plus chemotherapy”, “radiotherapy plus epidural steroid injection”, radiotherapy and radiosurgery alone were the least common modalities applied to treat LCH patients (2.5% each).

Among the multifocal and multisystem categories, “systemic chemotherapy” was the most frequent management approach applied (36.3% and 33.3%, respectively), followed by total excision in the multifocal cases and combination therapies (total excision + chemotherapy, subtotal excision + chemotherapy, chemotherapy + radiotherapy, and analgesics + intraregional steroid) in the multisystem group (18.2% and 44.4%, respectively). Two patients in the unifocal group and 1 patient in the multisystem and multifocal groups were treated by conservative management.

Following the initial treatments, 77.3% of the unifocal, 81.8% of the multifocal and 55.5% of the multisystem cases completely recovered, but permanent complications were reported in six patients (9.4%) (1 in the unifocal, 3 in the multifocal, and 2 in the multisystem group). The multisystem group had more recurrences than the unifocal and multifocal groups (11% vs 2% and 9%, respectively), and the mortality rate in this group was higher than that in the other two groups (11% vs 0.0%).

## Discussion

The results of this systematic review of rare case reports of LCH bone involvements showed differences and similarities with the existing literature. The cause may be that the available statistics are mostly based on studies concerning LCH in general and not rare cases. Furthermore, most of the findings of rare cases were obtained from a few cases, not systematic reviews, highlighting the need for these studies.

Our findings showed that most cases (70%) of rare LCH bone involvements occurred in male patients and the M/F ratio was 2.3. This finding was consistent with previous study findings [3, 6, 11]. However, unlike studies reporting that LCH disease is more prevalent in children, and rare in adults [2, 4, 6, 68], our findings showed no difference between the two groups [children = 31 cases (48.44%); adults = 33 cases (51.56%)].

Additionally, the mean age of the children in this study was 7.6 years, higher than that of children with the disease in other studies (3 years) [3]. The explanation for such differences is not clear but may indicate that most bone involvements of LCH occur at an older age or may be due to our inclusion of rare cases [4].

Our findings also revealed that the unifocal form (68.7%) was the most common form of bone involvement and the unifocal/multifocal ratio was 4:1. Singh et al. [63] also reported an average ratio of 3:1 (range 2:1 to 6:1) for monostotic-polyostotic lesions, and Tsuchie et al. [61] reported a high prevalence (79%) of solitary bone lesions.

Based on the available statistics, the skull is the most common site of bone involvement of LCH (40–60% of cases) [1, 11, 14, 61, 62, 67, 69], followed by the femur, rib, vertebra, and humerus in children [69] and isolated flat bone involvement in adults [1]. In only one study, the primary sites of bone involvement in adults were reported as follows: jaw (30%), skull (21%), extremity (17%), vertebra and pelvis (13% each), and rib (6%) [69].

The prevalence of bone involvements in children and adults showed the following differences: long bones (17%) with a higher tendency in children [1], jaw (8% in children, 30% in adults) [11, 69], pelvis (8%–13%) with a higher tendency in adults [1, 4, 69], ribs (8% in children, 6%–25% in adults) [1, 69], and spine (3%–30%) with a slightly higher tendency in children [57].

In the present study, the prevalence of bone involvement of LCH disease was as follows:

In children: skull (48.4%), chest wall (22.6%), spine and long bones (9.7% each); in adults: skull (36.4%), chest wall (21.2%), spine (9.1%) and jaw (6.1%). Consistent with previous studies [1, 9, 14, 61, 67, 69], our findings revealed that the skull was the most commonly involved site, and calvarium involvements were more common than the skull base.

Calvarium lesions were more common in children, and mostly involved the occipital and parietal regions. The skull base involvements mostly involved the clivus, sphenoid bone and inner ear and were more common in adults. The chest wall, particularly the clavicle and sternum, was the second most common site involved after the skull. We could not find exact data on clavicular involvement, but some studies have reported its incidence in LCH disease [7, 9, 37–39]. Consistent with the findings of these studies, we only found five cases of clavicular involvement. The sternum was the other rare location; to the best of our knowledge, only 13 cases of sternum LCH have been reported in the literature, and seven of them (six unifocal and one multifocal) were included in this study [43–45]. Scapular and humeral involvements were rare in this study, and our results showed only two case reports of scapular involvement, and one case of humeral involvement. Khung et al. [9] believed that “scapular lesions are not uncommon in children”, but Pandey et al. [47] reported the scapula as a rare location of LCH; based on our findings, the scapula seems to be a rare presentation of LCH. Kim et al. [69] reported the humerus as a frequent site in children, but our findings did not support their results; further studies are warranted in this area.

The forearms, metacarpals and metatarsals were the other rare sites found in our study, and they were previously reported to be rare or sporadic [2, 9, 14, 15].

As expected, localized pain and swelling or both were the most presenting signs in these patients; however, depending on the involved site and its severity, the symptoms were different. Our findings revealed the loss of consciousness with or without headache and vomiting due to extradural hematoma with LCH and myelopathy signs due to spinal cord compression, nerve palsy due to petrous apex involvement, and visual loss due to optic canal involvement, which are rare presentations of LCH [9, 12]. The presentation of LCH with extradural hematoma is an extremely rare presentation of the disease, and only ten cases have been reported in the literature [30]. We have identified nine of them in this review. Bleeding can be spontaneous and nontraumatic or traumatic. In cases with spontaneous bleeding, the exact etiology is unknown [30]. Four of the cases that we reviewed had a history of mild trauma, but the remainder did not.

Cord compression and neurologic deficits due to spinal involvement are extremely rare, and the threatening event of LCH usually present as localized or radiating pain, restricted mobility, numbness, weakness and paresthesia [31–35]. Khung et al. [9] reported that isolated cervical spine involvement is very rare, but Shaker et al. [57] believed that, based on the literature review, the cervical spine, compared with the thoracic or lumbar spine, has a higher prevalence. Therefore, for these conflicting results, we only included the pathological fracture or cord compression with spinal involvements. Additionally, involvement of the atlantoaxial joint, maxillary sinus, bilateral jaw, acetabulum, sacral spine (in adults), the crest and posterior of the ilium, and two cases of the femur involvement were reported as rare presentations of LCH [9, 50, 54, 56, 57, 63, 65–67] and were included in our results.

Consistent with previous studies, our findings revealed that imaging analyses, including plain radiograph, CT and MRI play crucial roles in the diagnosis of bone involvements. Based on the available study findings [9, 11, 13], most of the bone lesions were the osteolytic type. Additionally, intracranial hemorrhage, fluid–fluid levels, dura, intracranial extension, and pathological fractures were the rare radiologic features of LCH patients.

As mentioned previously, the treatment and outcome of bone lesions depend on their location, extent, and severity [6, 9]. Unifocal bone lesions have the best prognosis and are usually managed by observation or locally by excision, curettage, steroid injection or radiation therapy. Lesions that do not heal spontaneously or for which surgery is difficult require radiation or chemotherapy. By contrast, the multifocal and multisystem groups are usually treated using systemic chemotherapy [5, 11]. The current standard chemotherapy for the multifocal and low-risk multisystem groups is vinblastine and prednisone and mercaptopurine plus vinblastine and prednisone for the high-risk multisystem group. Clofarabine, cytarabine, etoposide and cladribine are salvage drugs [5, 70]. The multisystem group with high risk organ involvement has the worst prognosis [3].

Consistent with previous study findings [7, 49, 62], our findings revealed that local therapy and observation for the unifocal group, and systemic chemotherapy for the multifocal and multisystem groups were the most frequent management approaches applied. As expected, the recovery rates of the unifocal and multifocal groups were higher than that of the multisystem group, and the rate of recurrence and mortality in the multisystem group was higher than that in the other groups. Unexpectedly, our findings revealed that the recovery rate of the multifocal group (81.8) was higher than that of the unifocal group (77.3). The cause may be that, for the unifocal group, the follow-up data were not available for more patients compared with those for the multifocal group.

### Limitations

I. Given the incomplete data concerning patient follow-up in this review and lack of long-term follow-up for cases, we cannot make judgments regarding the long-term outcomes and reoccurrences of rare bone lesions of LCH in children or adults after treatment. Therefore, we suggest that future studies focus on exploring the long-term outcomes of treating “unusual sites of bone involvement in LCH patients”.

II. Additionally, our findings were derived from case reports, which rank at the bottom of the hierarchy of evidence. This inherent limitation in the study designs of the original articles covered in this systematic review restricts reaching a precise conclusion regarding the patient outcomes (i.e., rate of recovery, mortality rate, reoccurrence of symptoms, complications, and side effects).

## Conclusion

LCH is a rare disease that can affect any organ in the human body. The bone is the most commonly involved organ, and rare bone involvements may be the first or only symptoms of the disease. Because of the rarity of these lesions, their unfamiliarity may lead to misdiagnoses or delays in diagnosis. This review collected the clinical presentations, methods of diagnosis, radiologic features, management approaches, and outcomes of rare bone lesions of LCH in children and adults. Hopefully, our findings not only add to the body of literature concerning LCH but also provide some clues or guidance for physicians when encountering these cases.

## Data Availability

The datasets generated and analyzed for this study are not publicly available due to participant privacy but are available from the corresponding author upon reasonable request.

## Appendix

(“langerhans cell histiocytosis” OR “Hand-Schueller-Christian Syndrome” OR “Hand Schueller Christian Syndrome” OR “Hand Schüller Christian Disease” OR “Hand-Schüller-Christian Syndrome” OR “Hand Schüller Christian Syndrome” OR “Histiocytosis X” OR “Generalized Histiocytoses” OR “Generalized Histiocytosis” OR “Histiocytosis-X” OR “Langerhans Cell Histiocytoses” OR “Langerhans-Cell Granulomatosis” OR “Letterer-Siwe Disease” OR “Letterer Siwe Disease” OR “Non-Lipid Reticuloendotheliosis” OR “Non Lipid Reticuloendotheliosis” OR “Non-Lipid Reticuloendothelioses” OR “Schueller-Christian Disease” OR “Schueller Christian Disease” OR “Systemic Aleukemic Reticuloendotheliosis” OR “Systemic Aleukemic Reticuloendothelioses” OR “Type 2 Histiocytosis” OR “Type 2 Histiocytoses” OR “Langerhans Cell Granulomatosis” OR “Langerhans Cell Granulomatoses” OR “Hand-Schueller-Christian Disease” OR “Pulmonary Histiocytosis X” OR “Pulmonary Langerhans Cell Granulomatosis” OR “Hashimoto-Pritzger Disease”) AND (Head OR neck OR otorhinolaryngology OR Otology OR Laryngology OR Lung OR Skin OR Skull OR Bone OR Condyle OR Skin OR Lung OR Liver OR “Hematopoietic System” OR “Lymph Node” OR “Endocrine System” OR “Gastrointestinal System” OR “Central Nervous System” OR “Cerebrospinal Axis” OR “Cerebrospinal Axi”) AND (Pediatric OR Adult) AND (“Case report”)
